# Surgical Cases

**Published:** 1881-04

**Authors:** C. C. Frederick

**Affiliations:** House Physician


### BUFFALO GENERAL HOSPITAL.

SURGICAL CASES REPORTED BY DR. C. C. FREDERICK, HOUSE PHYSICIAN. (SERVICE OF DR. GAY.)

### RESECTION OF ELBOW JOINT.

CASE NO. 8375.

CHARLOTTE STRITTMEYER entered the hospital May 18, 1880; aged 28; married; German. Patient had necrosis of the elbow and also of the upper third of the femur, supposed to be congenital syphilis. At ten years of age, apparently a healthy child, she was afflicted with an abscess in the left hip, presumably not morbus coxaris, as she walked around upon it without much pain. Health constantly poor till the birth of her first child, when she was about twenty-two years of age. At that time the ulcer healed, she became fleshy and robust, and remained so till the birth of the second child, three and a half years after. She then failed again in health, the ulcer opened again, and one also broke out upon her left elbow, in which condition she entered the hospital, anæmic and very feeble.

She stated that her father, who is still living, has similar sores, and that a sister aged 19 died from the same. Her children are apparently healthy. She complains of no pain, and walks without limping. Dr. Gay ordered Tr. Cinchona Co. iron and cod-liver oil, nutritious diet and open air before using other constitutional treatment. Patient began to improve in health and strength, and June 15 ordered in addition to the above iodide of pot. gr. x three times daily. On June 23, 1880, Dr. Gay exsected the elbow joint and about three inches of the lower end humerus which was dead. July 7 put on an angular splint, and use passive motion, changing the angles from day to day. Aug. 1st, patient fleshy and strong, but discharge from elbow is offensive. Continued to discharge, and Oct. 20 probe showed dead bone, which was scraped out, and drainage tube inserted. Inflammation gradually subsided and wound nearly healed when patient left the hospital Dec. 1, 1880. Have not seen her since. Arm was movable at the elbow and of some use.

### RESECTION OF ELBOW JOINT.

### Case No. 8387.

Andrew Zeigler entered the hospital May 24, 1880; aged 9; born in America. Patient was hurt in Nov., 1879, at his elbow, while playing with his mates. He did not complain much about it at first, but in about six weeks pain and soreness on motion or pressure was noticed. Was seen by several physicians who manipulated the arm and added to its inflamed condition. On entrance Dr. Gay called consultation of the surgical staff. Conservatism was advised, hence the swollen tender arm was dressed in a plaster of Paris splint at right angles. The inflammation did not subside, neither was anchylosis under this treatment obtained, hence on June 29, 1880, Dr. Gay exsected the joint. July 8, put in angular splint and move at various angles from day to day. The wound healed rapidly, passive motion continued. All swelling and tenderness passed away and the patient was able to move his arm through quite a series of angles without pain. Discharged Jan. 7, 1881. Friends well pleased with the result.

### NECROSIS OF INFERIOR MAXILLA—EXSECTION. PLASTIC OPERATION.

### CASE NO. 8398.

CHARLES FILSTEAD entered June 5, 1880; aged 11 years; American. In Jan. 1880, patient had typho-malarial fever, in St. Louis, and following it an abscess on the left side of the inferior maxilla midway between the symphysis and angle. The tissues sloughed away exposing the greater portion of the jaw bone down to nearly its lower border, all the part exposed being necrosed.

June 16. Dr. Gay operated, assisted by Drs. Bartow, O'Brien and McBeth. The dead bone was found ready to separate from the living, and was removed readily. Dr. Gay then incised the skin of the upper lip on the left side and beneath the angle of the chin on the same side, thus enabling him to bring the skin in to fill up the opening. He then drew the parts together with silver wire, making a good-looking mouth. A great deal of inflammation set in, the tension was too great and the wire stitches tore out. Granulations then covered the remaining bone with flesh.

On Nov. 17, 1880, at a clinic before the medical class, Dr. E. M. Moore, of Rochester, performed a plastic operation upon the face, by moving a flap of skin from the neck up into the opening and retaining by means of hair lip pins. Removed the pins on the second day, nearly all united by first intention. In four weeks the face was entirely healed.

